# Novel algorithm for the diagnosis and treatment of different types of bile leakage following liver surgery

**DOI:** 10.1093/bjsopen/zrag110

**Published:** 2026-10-27

**Authors:** Sepehr Abbasi Dezfouli, Elmira Heidenreich, Mohammadamin Shahrbaf, Elias Khajeh, Ali Ramouz, Miriam Klauss, Katharina Hellbach, Osman Öcal, Martin Loos, Arianeb Mehrabi

**Affiliations:** Department of General, Visceral, and Transplantation Surgery, Heidelberg University Hospital, Heidelberg, Germany; Liver Cancer Centre Heidelberg (LCCH), Heidelberg University Hospital, Heidelberg, Germany; Department of General, Visceral, and Transplantation Surgery, Heidelberg University Hospital, Heidelberg, Germany; Department of General, Visceral, and Transplantation Surgery, Heidelberg University Hospital, Heidelberg, Germany; Department of General, Visceral, and Transplantation Surgery, Heidelberg University Hospital, Heidelberg, Germany; Department of General, Visceral, and Transplantation Surgery, Heidelberg University Hospital, Heidelberg, Germany; Department of Diagnostic and Interventional Radiology, Heidelberg University Hospital, Heidelberg, Germany; Department of Diagnostic and Interventional Radiology, Heidelberg University Hospital, Heidelberg, Germany; Department of Diagnostic and Interventional Radiology, Heidelberg University Hospital, Heidelberg, Germany; Department of General, Visceral, and Transplantation Surgery, Heidelberg University Hospital, Heidelberg, Germany; Department of General, Visceral, and Transplantation Surgery, Heidelberg University Hospital, Heidelberg, Germany; Liver Cancer Centre Heidelberg (LCCH), Heidelberg University Hospital, Heidelberg, Germany

**Keywords:** hepatectomy, bilioenteric anastomosis, posthepatectomy postoperative complications, treatment outcome

## Abstract

**Background:**

Posthepatectomy bile leakage (PHBL) after liver resection with bilioenteric anastomosis may originate from the anastomosis (anastomotic leak (AL)) or the transection surface (surface leak), including leakage from isolated bile ducts (IBDLs). Differentiating these sources remains challenging, and standardized management guidelines are lacking.

**Methods:**

All patients undergoing liver resection with bilioenteric anastomosis from 2008–2023 were analysed. PHBL was classified by origin (AL, surface leak, IBDL) and timing (early *versus* late onset). Drain fluid bilirubin concentration, drain-to-serum bilirubin ratio, and daily bilirubin loss were evaluated by leak type. Outcomes of conservative, interventional, and surgical management were compared.

**Results:**

Among 2936 liver resections, 229 involved bilioenteric anastomosis. PHBL occurred in 96 patients, including 44 ALs (46%), 47 surface leaks (49%), and 5 IBDLs (5%). AL showed higher peak drain bilirubin than surface leak/IBDL (38.3 *versus* 18.6 mg/dl; *P* < 0.001). Receiver operating characteristic analysis identified cut-offs for AL diagnosis: drain bilirubin > 20.85 mg/dl, drain-to-serum ratio > 10.57, and bilirubin loss > 160.93 mg/day. Management success varied by type and timing: AL and IBDL responded best to reoperation (success rates 78% and 100%), whereas surface leak was more effectively managed without surgery (65%). In AL specifically, reoperation was more successful in early-onset leaks (65% *versus* 38% in late-onset; *P* = 0.069), whereas interventional therapy was more successful in late-onset leaks (58% *versus* 15% in early-onset; *P* = 0.003).

**Conclusions:**

Distinct diagnostic thresholds help differentiate AL from other PHBL types. AL and IBDL benefit from reoperation, whereas surface leak is better managed without surgery. Timing influences therapeutic success, supporting a novel type- and timing-based management algorithm for PHBL.

## Teaser

Different types of posthepatectomy bile leakage require tailored treatment. This study proposes a diagnostic and therapeutic algorithm based on leak type and timing, enabling more effective management of bile leaks after liver resection with bilioenteric anastomosis.

## Introduction

Despite significant advancements in liver surgery, posthepatectomy bile leakage (PHBL) remains a major challenge. PHBL can lead to severe postoperative complications, prolonged hospital stays, and increased mortality^1,2^. It is often difficult to prevent and challenging to manage^3,4^. Although numerous studies have addressed PHBL, reported incidence rates and identified risk factors vary considerably across the literature^5,6^. Even after the introduction of a widely accepted definition and classification system by the International Study Group of Liver Surgery (ISGLS)^7^, discrepancies among studies persist.

One key reason for this inconsistency is that most studies, including that of the ISGLS, focus solely on the presence or absence of PHBL, without differentiating between types based on their anatomical source or underlying pathology^7^. Following liver resection with bilioenteric anastomosis (BEA), PHBL may originate from the anastomosis itself (anastomotic leak (AL)) or from the liver’s transection surface (surface leak (SL))^8^. These two forms represent distinct pathophysiological mechanisms and require separate consideration. SL can also result from isolated bile ducts (isolated bile duct leak (IBDL)), which are disconnected from the main biliary tree^8^. In many cases, accurately identifying the source of leakage remains challenging, as current diagnostic strategies are complex and sometimes inconclusive. Most studies fail to differentiate between leak types, limiting the reliability and applicability of their findings. As a result, clear guidelines for the diagnosis and treatment of PHBL remain scarce.

This study aims to address these gaps by analysing PHBL following liver resection with BEA over a 15-year period at a high-volume referral centre. The objectives were to classify PHBL types, develop diagnostic criteria to differentiate them, identify optimal treatment approaches based on anatomical origin and aetiology, and propose guidelines for clinical management.

## Materials and methods

### Study design

Data were collected from a prospectively maintained hepatectomy database. The study included all patients with hepatobiliary disorders who underwent major liver resection with concomitant BEA between January 2008 and April 2023. Approval was obtained from the regional ethics committee (approval number S-754/2018).

### Surgical procedures

Major hepatectomy was defined as the resection of four or more segments, according to the Brisbane 2000 terminology^9,10^. Hepatic resection was conducted using different techniques (Endovascular stapler, LigaSure, Cavitron Ultrasonic Surgical Aspirator, or Crush-Clamp). BEA was performed in a standardized fashion as an end-to-side anastomosis to a retrocolic Roux-en-Y limb, using an interrupted single-layer technique with polydioxanone 5-0 or 6-0 sutures^11^. In cases with multiple intrahepatic bile ducts, ducts were either combined into a common ostium or anastomosed individually to the jejunal limb. A surgical drain was placed adjacent to all anastomoses. No transanastomotic stents were used.

### Bile leak diagnosis and origin

Fluid from the surgical drains placed during surgery was routinely analysed for bilirubin, with standard assessment performed on postoperative day (POD) 3. However, if the drain output appeared suspicious in colour or volume, earlier analysis was undertaken. Drains were removed once no evidence of bile leakage or bleeding was observed. Bile leakage was diagnosed and graded according to the ISGLS definition for bile leakage after hepato-pancreato-biliary (HPB) surgery^7^. In patients whose drains had already been removed, a bile leak was suspected in the presence of symptoms such as fever, elevated inflammatory markers, abdominal pain, nausea, or vomiting. In such cases, ultrasound and further imaging were performed. If a fluid collection was detected, a percutaneous drain was inserted under computed tomography (CT) guidance, and the fluid was analysed for bilirubin.

The exact origin of bile leakage was identified using various diagnostic methods. In patients with a CT-guided drain in place, contrast agent was injected through the drain, followed by fluoroscopy. If the bile ducts and the jejunal loop were visualized, AL was diagnosed; otherwise, SL was assumed.

In patients who underwent percutaneous transhepatic cholangiodrainage (PTCD), contrast medium was injected through the PTCD catheter. Extravasation at the level of the anastomosis confirmed AL (*Fig. S1*), whereas leakage from the liver surface indicated SL (*Fig. S2*). If no extravasation was observed, magnetic resonance cholangiopancreatography or hepatobiliary-phase magnetic resonance imaging (MRI) using Primovist was performed. In cases of IBDL, contrast extravasation from a segmental bile duct without communication with the central biliary tree was observed (*Fig. S3*).

In patients who required reoperation, the diagnosis was either established or confirmed during surgery.

### Early and late bile leakage

Based on the timing of diagnosis, bile leaks were categorized as early or late. Early bile leakage was defined as leakage diagnosed before POD 7, whereas leakage identified on or after POD 7 was considered late. This distinction was made because early bile leakage is typically attributed to technical factors such as inadequate closure techniques or flaws in the anastomosis^12^. In contrast, late bile leakage is more often associated with patient-related factors including compromised blood supply, ischaemia, or impaired tissue healing^13^. Treatment options may vary depending on the timing of the leak, as reoperation after POD 7 is more challenging due to the development of adhesions, peritonitis, and the patient's critical condition^12,13^.

### Bile leak management

The management of bile leakage was based on the timing and severity of the leak, as well as the patient’s clinical condition. Initial treatment was conservative, involving observation, antibiotics, and maintenance of drains. If imaging showed inadequate drainage of intraabdominal collections, CT-guided drain insertion was performed. If the patient´s condition worsened despite adequate drainage (whether surgical or CT-guided drainage) or if bile output increased, further intervention or reoperation was considered. If first-line therapy failed, the alternative treatment was administered. Successful therapy was defined as the resolution of bile leakage (ISGLS criteria for bile leakage were no longer met, with no radiologic signs of leakage and no symptoms suggesting a bile leak). Persistent leakage at the time of death was considered therapy failure.

### Data collection

Patient demographic data were obtained before surgery. Intraoperative data included the indication for surgery, extent of liver resection, number of anastomoses, blood loss, and operation time. Postoperative evaluations assessed posthepatectomy haemorrhage (PHH), PHBL, and posthepatectomy liver failure (PHLF), all according to ISGLS definitions^14^. Major complications (Clavien–Dindo classification ≥ 3^15^), the length of intensive care unit (ICU) and hospital stay, in-hospital mortality, and the occurrence of biliary stenosis were also recorded. The management of PHBL was documented, as well as drain fluid data, such as drain bilirubin concentration, serum bilirubin, and drain volume. Drain bilirubin/serum bilirubin ratio and the net weight of bilirubin loss/day (drain bilirubin × volume of drain/100) were calculated.

### Statistical analysis

Statistical analyses were performed using SPSS, version 29. Continuous variables are presented as mean(standard deviation (s.d.)) or median (Q1, Q3), and categorical variables are reported as absolute numbers and percentages. Bivariate analysis was conducted using the Mann–Whitney *U* test for continuous variables, and the χ^2^ or Fisher’s exact test for categorical variables. Cut-off values were determined through receiver operating characteristic (ROC) curve analysis. Statistical significance was defined as *P* < 0.050.

## Results

### Preoperative and intraoperative data

During the 15-year study period, 2936 patients underwent liver resection. Among them, 229 patients (7.8%) underwent major hepatectomy with concomitant BEA. Of these, 96 patients (41.9%) developed PHBL and were included in the study. SL was observed in 52 patients (54%) and AL in 44 (46%). Some of the data from these 44 patients have been previously published^16^. *Table 1* summarizes the pre-, intra-, and postoperative data of patients with PHBL. No significant differences were observed between the two leakage groups in demographics (age, gender, body mass index, American Society of Anaesthesiologists score, prevalence of diabetes), laboratory data, tumour characteristics, or intraoperative data (extent of hepatectomy, number of anastomoses, blood loss, operation time).

**Table 1:** Preoperative, intraoperative and postoperative characteristics of patients undergoing liver resection with BEA, overall and by PHBL type

	Patients with LR + BEA (*n* = 229)	PHBL (*n* = 96)	SL/IBDL (*n* = 52)	AL (*n* = 44)	P*
**Demographic data**					
Age (years), mean(s.d.)	63(12.2)	66(11)	64(13)	69(9)	0.126
Sex					
Male	143 (62.4%)	63 (66%)	38 (73%)	25 (57%)	0.095
Female	86 (37.6%)	33 (34%)	14 (27%)	19 (43%)	
BMI (kg/m^2^), mean(s.d.)	25.8(5.5)	26.3(6.9)	26(4.4)	26.8(9.1)	0.750
ASA ≥ 3	110 (48.0%)	46 (48%)	22 (42%)	24 (55%)	0.232
Diabetes	26 (11.4%)	16 (17%)	8 (15%)	8 (18%)	0.714
**Laboratory data**					
AST (U/l), mean(s.d.)	102(173)	92(87)	96(93)	86(79)	0.670
ALT (U/l), mean(s.d.)	127(204)	115(128)	123(142)	106(110)	0.476
GGT (U/l), mean(s.d.)	567(551)	581(534)	625(575)	530(485)	0.521
Bilirubin total (mg/dL), mean(s.d.)	3.4(4.0)	3.1(3.7)	2.7(3.2)	3.4(4.1)	0.899
Bilirubin direct (mg/dl), mean(s.d.)	2.5(3.6)	1.7(2.8)	1.6(2.5)	2(3.3)	0.892
**Tumour characteristics**					
Cholangiocarcinoma	187 (81.7%)	84 (88%)	46 (89%)	38 (86%)	0.550
Gallbladder carcinoma	14 (6.1%)	4 (4%)	2 (4%)	2 (5%)	
Metastasis	12 (5.2%)	4 (4%)	3 (6%)	1 (2%)	
Other	16 (7.0%)	4 (4%)	1 (2%)	3 (7%)	
**Intraoperative data**					
Left hemihepatectomy	84 (36.7%)	40 (42%)	20 (39%)	20 (46%)	0.489
Right hemihepatectomy	145 (63.3%)	56 (58%)	32 (62%)	24 (55%)	
Extended hepatectomy	117 (51.1%)	40 (42%)	24 (46%)	16 (36%)	0.332
Hemihepatectomy	112 (48.9%)	56 (58%)	28 (54%)	28 (64%)	
1 Anastomosis	197 (86.0%)	83 (87%)	45 (87%)	38 (86%)	0.818
> 1 Anastomoses	32 (14.0%)	13 (14%)	7 (14%)	6 (14%)	
Blood loss (ml), mean(s.d.)	1951(1380)	1839(1219)	1790(1130)	1897(1327)	0.794
Duration of operation (min), mean(s.d.)	362(99)	362(104)	365(111)	359(96)	0.695
**Postoperative data**					
PHH	15 (6.6%)	12 (13%)	5 (10%)	7 (16%)	0.353
PHBL grade					
A	/	3 (3%)	3 (6%)	0 (0%)	< 0.001
B	/	51 (53%)	35 (67%)	16 (36%)	
C	/	42 (44%)	14 (27%)	28 (64%)	
PHLF	42 (18.3%)	18 (19%)	9 (17.3%)	9 (21%)	0.694
Clavien-Dindo Class ≥ 3b	80 (34.9%)	48 (50%)	20 (39%)	28 (64%)	0.014
Length of ICU stay (days), mean(s.d.)	14(18)	16(20)	12(14)	22(24)	0.010
Length of hospital stay (days), mean(s.d.)	38(25)	47(25)	43(19)	52(30)	0.229
All-cause mortality	45 (19.7%)	22 (23%)	7 (14%)	15 (34%)	0.017
Biliary stenosis	13 (5.7%)	11 (12%)	4 (8%)	7 (16%)	0.208

Values are *n* (%) unless otherwise stated. LR, liver resection; BEA, bilioenteric anastomosis; PHBL, posthepatectomy bile leakage; SL, surface leak; IBDL, isolated bile duct leak; AL, anastomotic leak; BMI, body mass index; ASA, American Society of Anaesthesiologists; AST, aspartate aminotransferase; ALT, alanine aminotransferase; GGT, gamma-glutamyl transferase. PHH, post hepatectomy haemorrhage; PHLF, post hepatectomy liver failure; ICU, intensive care unit; min, minute. *Comparison between SL/IBDL and ABL. χ^2^ test or Fisher’s exact test were used for categorical variables, Mann–Whitney *U* test was used for continuous variables. / Indicates not applicable.

The mean(s.d.) age of patients with PHBL was 66(11) years, and 63 patients (66%) were male. Cholangiocarcinoma was the predominant tumour type (84 cases, 88%), followed by gallbladder carcinoma, metastases (colorectal and pancreatic primaries) and other conditions (echinococcosis and intraductal papillary neoplasm of the bile duct), each with 4 cases (4%). The most common procedure was right hemihepatectomy, performed in 56 patients (58%). In most cases, a single BEA was performed (83 patients, 87%).

### Postoperative data

The occurrence of PHH was similar in both groups (5 of 52, 10% *versus* 7 of 44, 16%; *P* = 0.353). Patients with AL had a significantly higher PHBL grade. Most patients with AL (28 of 44, 64%) had PHBL grade C, whereas the majority of patients with SL (35 of 52, 67%) had PHBL grade B (*P* < 0.001). PHLF was similar in both groups (9 of 52, 17% *versus* 9 of 44, 21%; *P* = 0.694). Compared with patients with SL, the AL group had a significantly higher percentage of patients with Clavien–Dindo classification ≥ 3b complications (20 of 52, 39% *versus* 28 of 44, 64%; *P* = 0.014). Patients with AL experienced significantly longer ICU stays (12 days *versus* 22 days; *P* = 0.010). The length of hospital stay was similar in both groups (43 days *versus* 52 days; *P* = 0.229). In-hospital mortality was significantly higher in the AL group (7 of 52, 14% *versus* 15 of 44, 34%; *P* = 0.017). There was no significant difference in the occurrence of biliary stenosis between the two groups (4 of 52, 8% *versus* 7 of 44, 16%; *P* = 0.208).

### Drain fluid analysis

Drain fluid characteristics are presented in *Table 2*. Patients with AL showed significantly higher peak bilirubin concentration in their drain fluid compared with those with SL (38.3 mg/dl *versus* 18.6 mg/dl; *P* < 0.001). Similarly, the ratio of drain-to-serum bilirubin concentration (measured on the day of peak drain bilirubin) was significantly higher in the AL group (13.8 *versus* 9.5; *P* = 0.039). Drain volume was higher in the SL group, but the difference was not statistically significant (577.5 ml *versus* 800 ml; *P* = 0.154). The net weight of bilirubin loss per day (calculated as drain bilirubin concentration × drain volume/100) was higher in the AL group, although the difference was not significant (202.4 mg *versus* 144.4 mg; *P* = 0.093).

**Table 2:** Diagnostic value of drain fluid parameters in patients with PHBL

	PHBL (*n* = 96)	SL/IBDL (*n* = 52)	AL (*n* = 44)	P*	Cut-off point	Sensitivity; Specificity	LR+; LR−	AUC†	Youden’s index
Peak drain bilirubin (mg/dl)	24.9 (14.2, 44.8)	18.6 (11.6, 25.4)	38.3 (21.2, 55.3)	<0.001	20.85	73%; 67%	2.21; 0.40	0.75 (0.65, 0.85)	0.40
Drain bilirubin/serum bilirubin	11.5 (6.5, 29.9)	9.5 (5.4, 20.1)	13.8 (9.1, 35.9)	0.039	10.57	73%; 60%	1.83; 0.45	0.62 (0.51, 0.74)	0.32
Volume of drain (ml)	700 (400, 1350)	800 (500, 1400)	577.5 (330, 1300)	0.154					
Net bilirubin loss/day (mg)‡	173.4 (96.9, 316.5)	144.4 (76.8, 240.6)	202.4 (110.9, 378.8)	0.093	160.93	67%; 61%	1.72; 0.54	0.62 (0.48, 0.75)	0.28

Values are median (i.q.r.) unless otherwise stated. †Values in parentheses are 95% confidence intervals. PHBL, posthepatectomy bile leakage; SL, surface leak; IBDL, isolated bile duct leak; AL, anastomotic leak; LR+, positive likelihood ratio; LR−, negative likelihood ratio; AUC, area under the curve. *P values were calculated using the Mann–Whitney *U* test. ‡Net weight of bilirubin loss/day = drain bilirubin × volume of drain/100.

Results of the ROC curve analysis for determining cut-off values to detect AL in patients with PHBL are summarized in *Table 2*. The corresponding ROC curves are provided in *Fig. 1*. ROC curve analysis identified an optimal cut-off for a peak drain bilirubin concentration of 20.85 mg/dl, with an area under ROC curve (AUC) of 0.75 and a sensitivity and specificity of 73% and 67%, respectively. For the drain bilirubin-to-serum bilirubin ratio, the optimal cut-off was 10.57, with an AUC of 0.62, a sensitivity of 73%, and a specificity of 60%. For the net weight of bilirubin loss per day (mg), a cut-off point of 160.93 mg was identified, with a sensitivity of 67% and a specificity of 61%.

**Figure 1:**
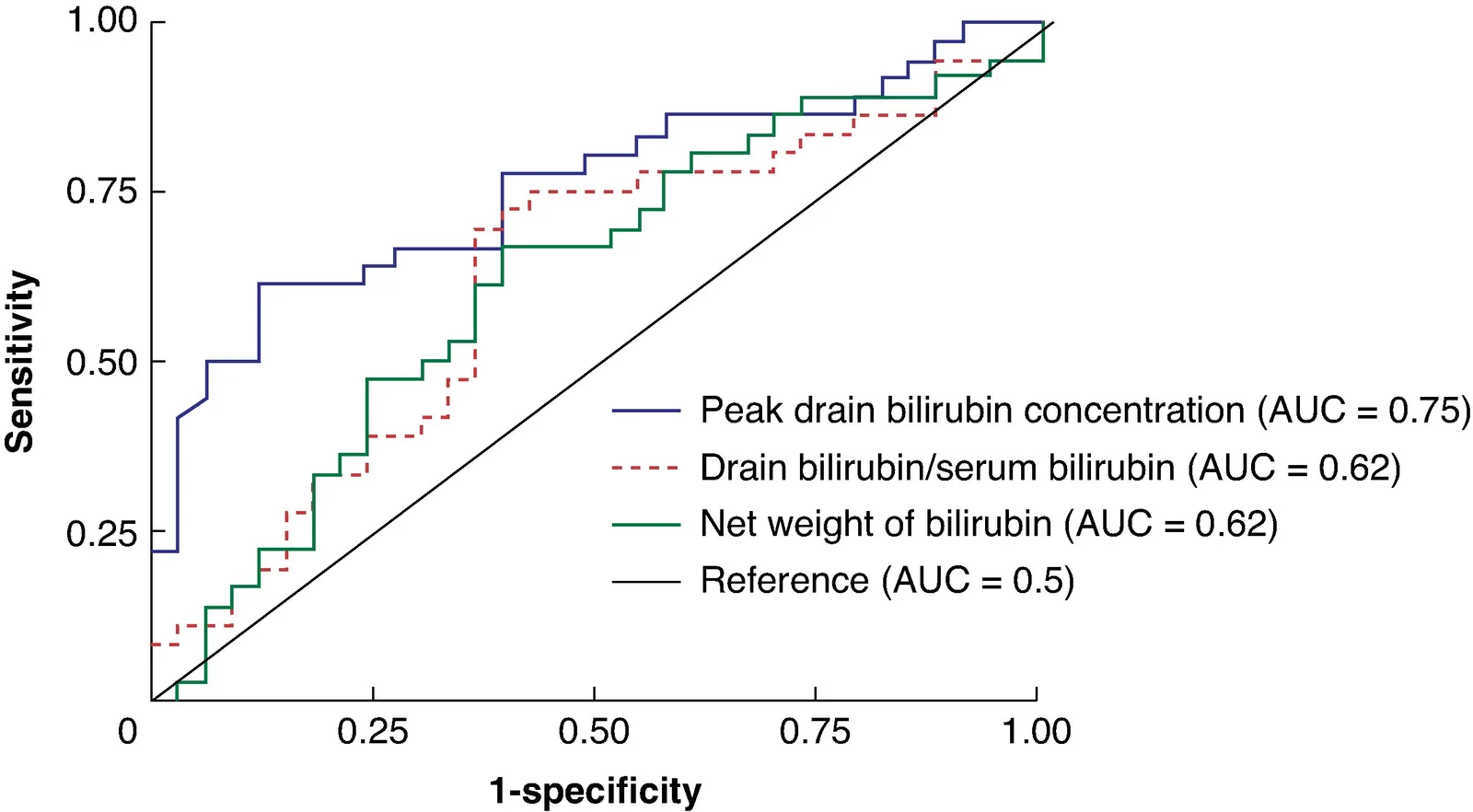
ROC curves for diagnostic cut-offs to detect AL in patients with PHBL PHBL, posthepatectomy bile leak; AUC, area under the curve.

### Management of bile leakage

*Figure 2* presents the treatment flowchart for patients with PHBL. Of the 96 patients initially treated conservatively, success was achieved in 26 of 47 (55%) cases with SL but in none of the AL (0 of 44, 0%) or IBDL (0 of 5, 0%) cases (*P* < 0.001). The remaining 70 patients required additional treatments including PTCD (36) and reoperation (34) after failed conservative management.

**Figure 2:**
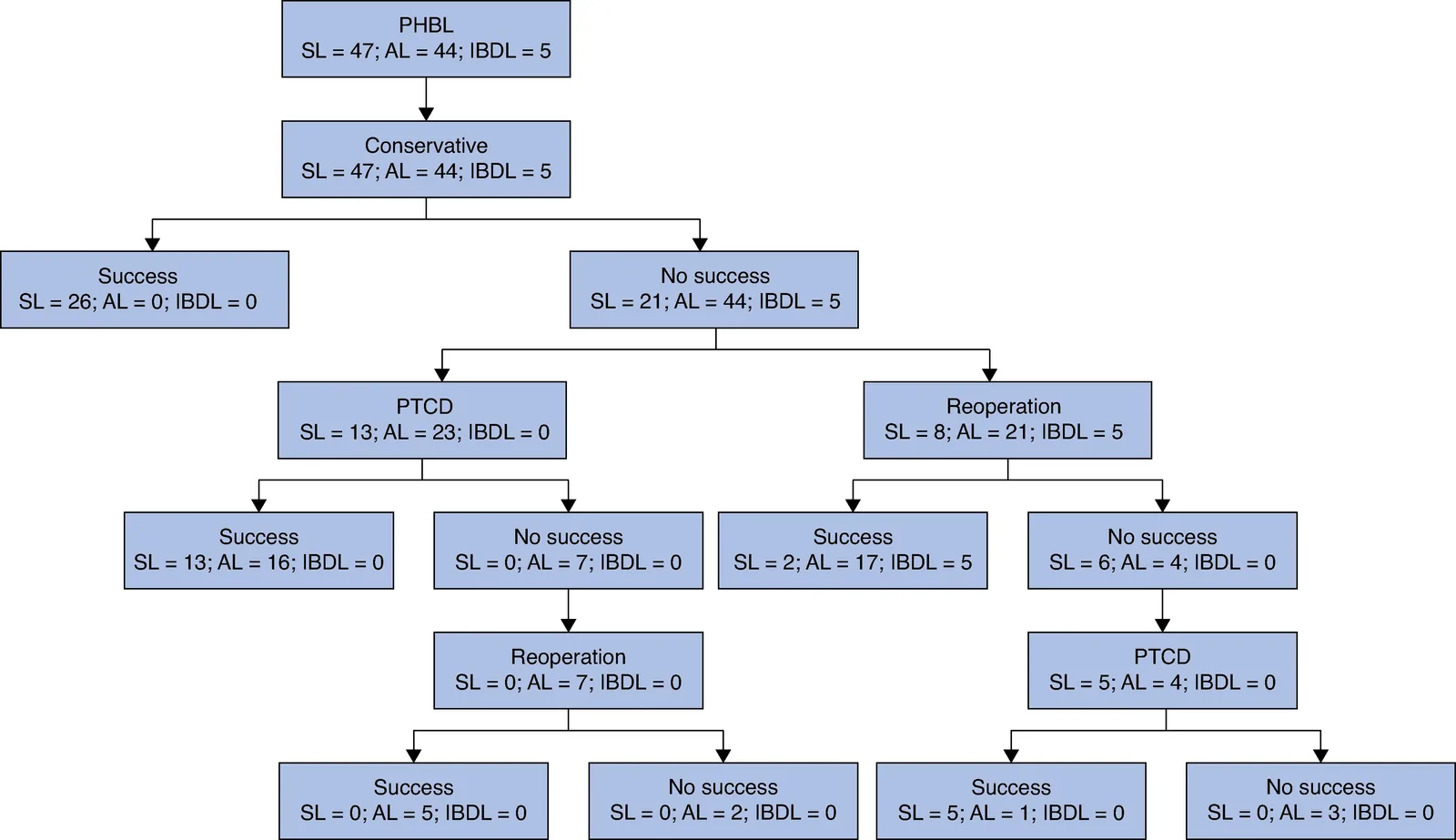
Performed treatments and outcomes in patients with PHBL PHBL, posthepatectomy bile leakage; SL, surface leak; AL, anastomotic leak; IBDL, isolated bile duct leak; PTCD, percutaneous transhepatic cholangiography and drainage.

PTCD was successful in 13 of 13 (100%) SL cases and 16 of 23 (70%) AL cases (*P* = 0.070). The remaining 7 patients with AL underwent reoperation, which was successful in 5 of 7 (71%) cases. Reoperation was successful in 2 of 8 (25%) SL, 17 of 21 (81%) AL, and 5 of 5 (100%) IBDL cases (*P* = 0.002). Of the remaining 10 patients, 5 with SL and 4 with AL underwent PTCD. This was successful in 5 of 5 (100%) patients with SL but only in 1 of 4 (25%) with AL (*P* = 0.033).

Despite these interventions, 6 patients died with unresolved bile leakage, including 1 of 52 (2%) with SL and 5 of 44 (11%) with AL (*P* = 0.160).

The overall success rates of different treatment strategies by leak type are compared in *Fig. 3*. For SL, PTCD was most effective (18 of 18, 100%), followed by conservative management (26 of 47, 55%), and reoperation (2 of 8, 25%), with significant differences (*P* < 0.001). For AL, reoperation showed the highest success rate (22 of 28, 79%), followed by PTCD (17 of 27, 63%), whereas conservative management was unsuccessful in all cases (0 of 44, 0%), also with significant differences (*P* < 0.001). In patients with IBDL, only reoperation was successful (5 of 5, 100%); conservative treatment failed in all (0 of 5, 0%), whereas PTCD was not administered in any patient (*P* = 0.008).

**Figure 3:**
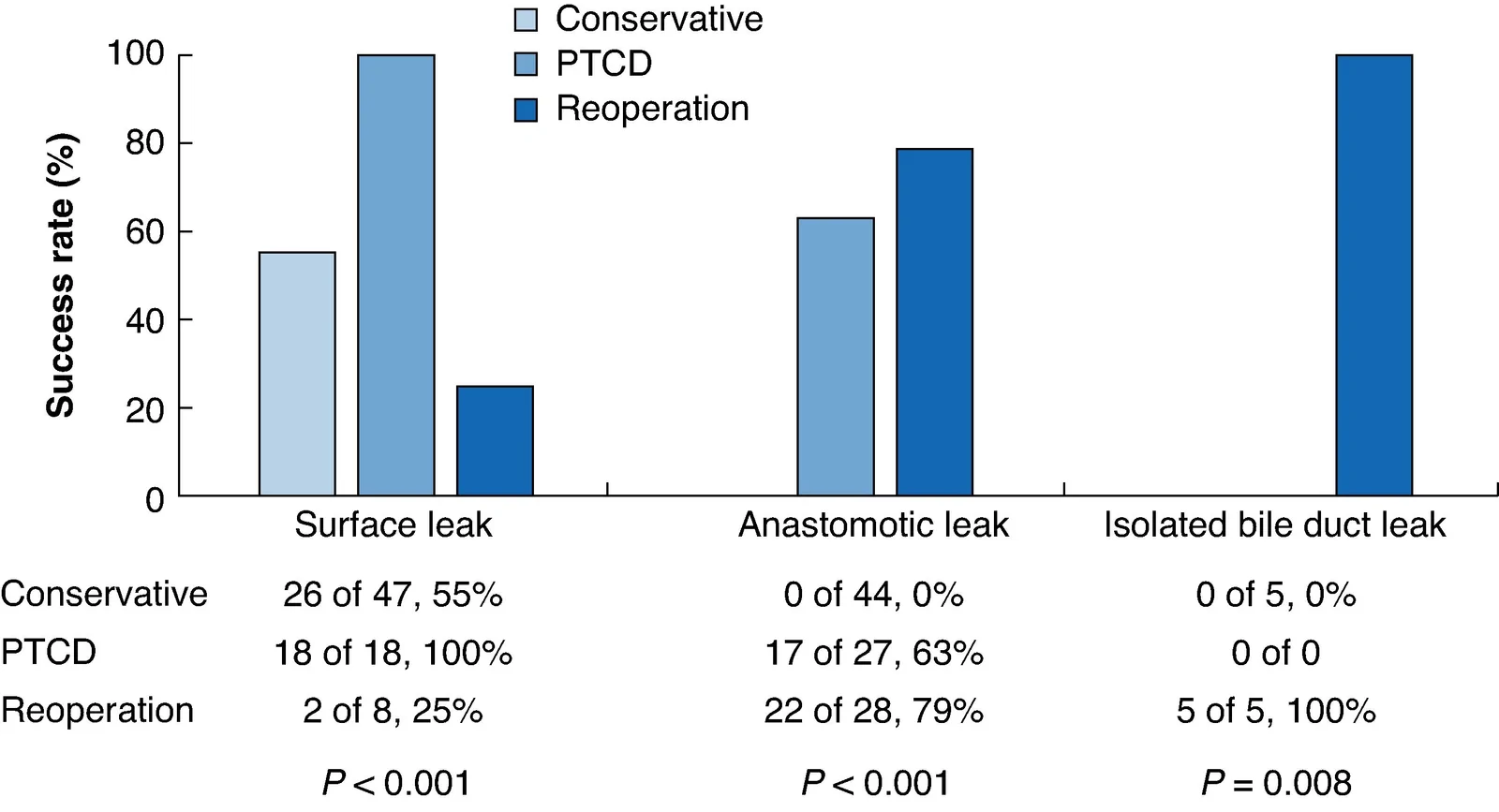
Success rates of different treatment strategies for patients with PHBL In 70 of 96 patients more than one treatment was administered. PHBL, posthepatectomy bile leak; PTCD, percutaneous transhepatic cholangiography and drainage.

### Early *versus* late bile leakage

*Table 3* compares treatment outcomes in early *versus* late PHBL. Of the 96 patients, 39 had early leakage (diagnosed before POD 7), whereas 57 had late leakage (diagnosed on or after POD 7).

**Table 3:** Successful treatment of early *versus* late bile leakage

Successful treatment	PHBL (*n* = 96)	Early leakage (< POD 7 (*n* = 39)	Late leakage (≥ POD 7) (*n* = 57)	P*
**SL/IBDL**				
Conservative	26 (50%)	8 (42%)	18 (55%)	0.388
PTCD	18 (35%)	7 (37%)	11 (33%)	0.798
Reoperation	7 (14%)	4 (21%)	3 (9%)	0.224
None	1 (2%)	0 (0%)	1 (3%)	0.444
**AL**				
Conservative	0 (0%)	0 (0%)	0 (0%)	/
PTCD	17 (39%)	3 (15%)	14 (58%)	0.003
Reoperation	22 (50%)	13 (65%)	9 (38%)	0.069
None	5 (11%)	4 (20%)	1 (4%)	0.160

Values are *n* (%). PHBL, posthepatectomy bile leakage; SL, surface leak; IBDL, isolated bile duct leak; AL, anastomotic leak; PTCD, percutaneous transhepatic cholangiography and drainage. *χ^2^ test or Fisher’s exact test were used for categorical variables, Mann–Whitney *U* test was used for continuous variables.

In patients with SL, there was no statistically significant difference in successful treatment between patients with early and late leakage. Conservative treatment and PTCD were generally the most successful treatments, regardless of timing of the leakage. With AL, PTCD was significantly more often the successful treatment in late leakage (3 of 39, 15% *versus* 14 of 57, 58%; *P* = 0.003). Reoperation was more successful in early leakage (13 of 39, 65% *versus* 9 of 57, 38%; *P* = 0.069), although this difference was not statistically significant.

## Discussion

In this single-centre experience including 2936 patients, major liver resection with concomitant BEA was performed in 229 cases. PHBL occurred in 44 patients (19.2%) from the BEA, 47 patients (20.5%) from the transection surface, and 5 patients (2.1%) from isolated bile ducts. Patients with AL experienced significantly higher rates of major complications, longer ICU and hospital stays, and higher mortality rates. The mean peak drain bilirubin concentration in patients with AL was significantly higher compared with those with SL (38.3 mg/dl *versus* 18.6 mg/dl, respectively; *P* < 0.001). In patients with SL, interventional and conservative therapies showed better success rates (100% and 81%, respectively, *versus* 22% after reoperation). In contrast, AL leaks responded better to reoperation (82% *versus* 63% with interventional therapy and 0% with conservative therapy). In cases of IBDL, only reoperation was successful (100% *versus* 0% after other therapies).

Studies analysing bile leakage after liver resection with BEA are scarce and mostly limited to small series, with reported leak rates ranging from 3.6% to 50%^17-19^. A major strength of the present study lies in the inclusion of the largest cohort of patients with PHBL in liver surgery with BEA and its detailed characterization of diagnostic and therapeutic strategies across different leak types.

Planning proper treatment strategies for PHBL requires a thorough understanding of its aetiology. Therefore, each PHBL type should be considered separately. Differentiating between PHBL types is challenging and requires detailed diagnostic evaluation^20^. This appears to be the only high-volume study evaluating all three PHBL types separately within the same cohort. AL was associated with significantly longer ICU stays and higher rates of major complications and mortality, indicating a more severe clinical entity. Early diagnosis and prompt initiation of appropriate therapy are crucial, as they directly influence therapeutic decision-making and may improve patient outcomes. To enable early identification, a novel approach based on drain bilirubin concentration is proposed. ROC analysis indicated that a drain bilirubin concentration > 20 mg/dl or a drain-to-serum bilirubin ratio > 10 are sensitive for diagnosing AL. This approach enables earlier and more straightforward differentiation between PHBL types, facilitating prompt therapeutic decisions. To account for dilutional effects from ascites, the total daily bilirubin loss (net weight) was calculated and compared between leak types. ROC analysis indicated a loss of more than 160 mg/day was predictive of AL.

Therapy success was then compared across different treatment modalities for each PHBL type. Non-surgical management of SL was successful in 85% of cases (50% conservative and 35% PTCD), whereas reoperation had a success rate of only 22%. SL typically arises from small peripheral bile ducts that tend to close spontaneously^21^. In these cases, PTCD likely facilitates bile drainage by reducing resistance in the central biliary system and stenting the anastomosis, thereby promoting healing^22,23^. Surgery, however, often fails to address the underlying issue, as clipping or suturing the liver parenchyma is usually not helpful^23,24^. The only subgroup of SL patients benefiting from reoperation were those with IBDL, in whom all other therapies failed, and surgical treatment was successful in all cases (5 of 5, 100%). IBDL does not resolve spontaneously unless the corresponding segment atrophies or is resected^8,24^. In the present cohort, the isolated segment was either resected or an additional BEA was performed.

Even within each PHBL type, different underlying aetiologies may exist. PHBL can result from technical failure of the anastomosis or transection surface closure (typically leading to early leaks), as well as impaired healing due to ischaemia or sepsis (more often associated with late leaks). In most cases, however, the exact cause remained unclear, precluding a direct correlation between aetiology and therapy success. Therefore, outcomes were analysed according to timing of PHBL diagnosis.

Late-diagnosed leaks (≥ POD 7) were associated with worse prognosis, including longer hospital stay (51 *versus* 41 days), longer ICU stays (20 *versus* 11 days), and a higher, though non-significant, mortality rate (26% *versus* 18%). With SL, treatment success was not influenced by timing. With AL, reoperation was more successful in early PHBL (65% *versus* 35%), whereas PTCD was more effective in late-onset PHBL (58% versus 15%).

Currently, there are no universally accepted guidelines for managing bile leakage after HPB surgery. Based on these findings, a novel algorithm is proposed (*Fig. 4*). After PHBL diagnosis, low-flow bile leakage, defined as drain/serum bilirubin ratio < 10, drain bilirubin concentration < 20 mg/dl, and a net weight of bilirubin loss < 160 mg/day, is initially considered as transection surface leakage and managed conservatively. If low-flow leakage persists, PTCD is recommended. High-flow bile leakage, defined as any of these thresholds exceeding the above cut-offs, is considered suggestive of BEA leakage and managed according to timing. Early leakage (< POD 7) should undergo reoperation as first-line therapy. For patients with late leakage (≥ POD 7), PTCD is recommended as the initial treatment. If first-line therapy fails, the alternative approach should be employed. Patients deteriorating under conservative management or not responding to PTCD for low-flow leakage should be escalated to the high-flow pathway.

**Figure 4:**
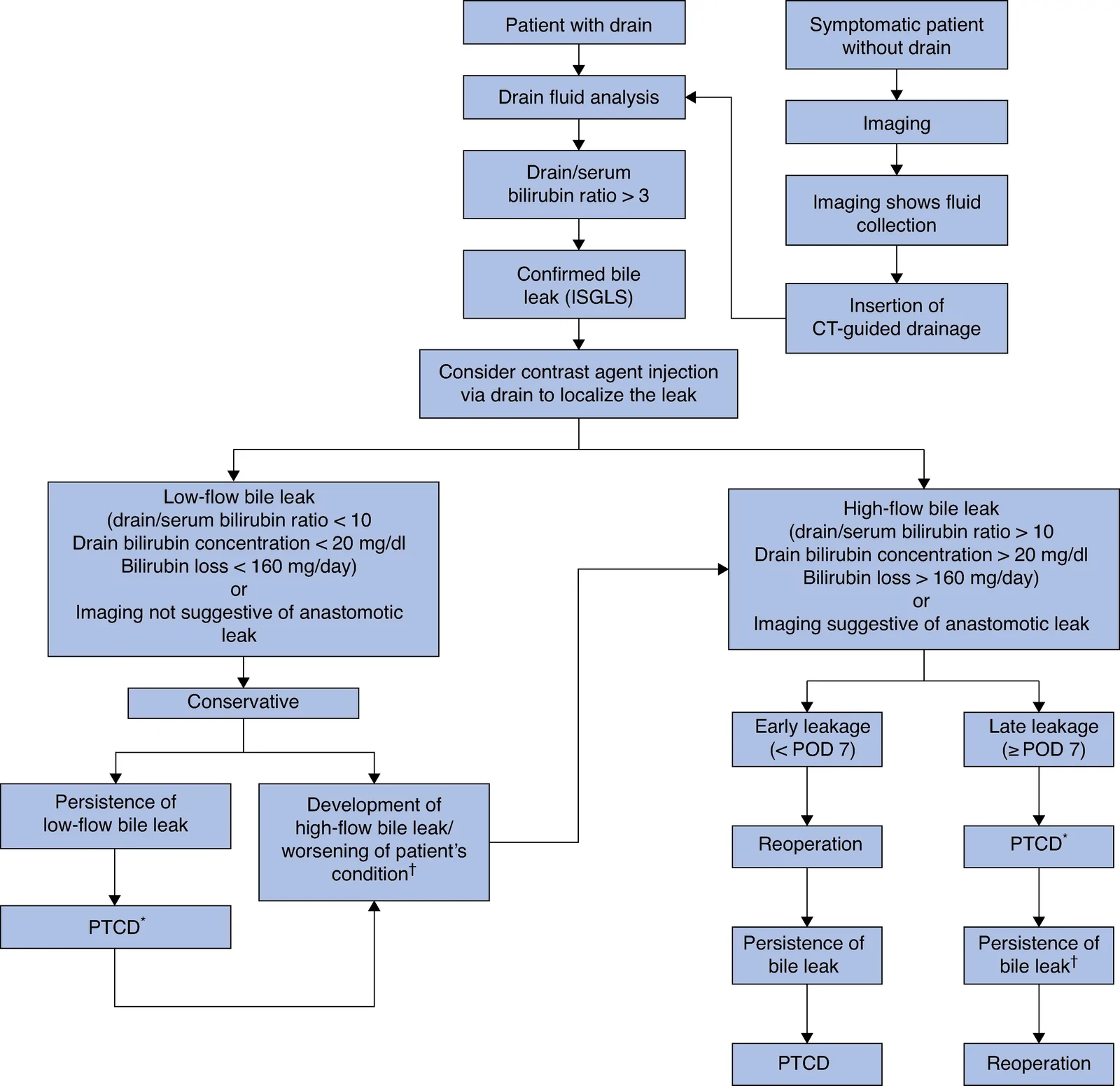
Proposed diagnosis and treatment algorithm for patients with PHBL after liver resection with BEA *Consider contrast injection via PTCD to localize the leak. †Consider Primovist-enhanced MRI to exclude isolated bile duct leak. PHBL, posthepatectomy bile leak; BEA, bilioenteric anastomosis; MRI, magnetic resonance imaging; ISGLS, International Study Group of Liver Surgery; CT, computed tomography; PTCD, percutaneous transhepatic cholangiography and drainage; POD, postoperative day.

In cases of persistent leakage or suspected anastomotic origin, contrast injection via drains or PTCD should be performed to localize the leak. If localization is not achieved, Primovist-enhanced MRI may be considered to exclude IBDL.

Drain bilirubin analysis represents a simple and rapid tool that may help early identification of AL, albeit with moderate sensitivity and specificity (73% and 67%, respectively). Further diagnostic investigations should be performed, when feasible, to confirm the diagnosis.

Individual decision-making remains crucial, but this algorithm is the first to include general recommendations for PHBL and has the potential to help standardize treatment across institutions.

This study has several limitations. First, its retrospective design means that the proposed diagnostic and therapeutic algorithm was developed based on past clinical practice; consequently, not all patients in the cohort were managed according to this algorithm. Second, the long study period introduces the possibility of chronological bias. Although surgical techniques and standards remained largely unchanged, improvements in perioperative management and increasing institutional experience may have influenced diagnostic accuracy and treatment outcomes. The cohort comprised patients with advanced tumours undergoing major hepatectomy with BEA, resulting in a relatively high mortality rate of 19.7%. Although the retrospective design precluded precise determination of the cause of death, mortality was likely attributable to liver failure and/or septic complications related to bile leakage. Finally, the single-centre design and the complexity of the proposed diagnostic and therapeutic strategies may limit their generalizability and reproducibility in non-tertiary hospitals, as their implementation requires a high level of expertise, especially in interventional and diagnostic radiology.

In conclusion, PHBL after liver surgery with BEA should be classified into BEA leaks, leaks from the transection surface, and leaks from isolated bile ducts. Patients with BEA leaks show a worse prognosis, with higher rates of severe complications and mortality. A drain bilirubin level greater than 10.5 times the serum bilirubin concentration is highly indicative of BEA leakage. BEA leaks and leaks from isolated bile ducts respond better to reoperation, whereas transection SLs are best managed conservatively. A novel diagnostic and therapeutic algorithm based on leak type and timing is proposed.

## Supplementary Material

zrag110_Supplementary_Data

## Data Availability

The data relevant to this study are available from the corresponding author on request.
