# Supplementary material for: Novel algorithm for the diagnosis and treatment of different types of bile leakage following liver surgery
Source: BJS Open. 2026 Oct 27;10(5):zrag110. doi: 10.1093/bjsopen/zrag110 (PMC13711662; doi:10.1093/bjsopen/zrag110)
Supplement: Supplementary file 1 [file zrag110_supplementary_data.docx]

**A Novel Algorithm for the Diagnosis and Treatment of Different Types of Bile Leakage Following Liver Surgery**

Sepehr Abbasi Dezfouli^1,2^*, MD; Elmira Heidenreich^1^*; Mohammadamin Shahrbaf^1^, MD; Elias Khajeh^1^, MD; Ali Ramouz^1^, MD; Miriam Klauss^3^, MD; Katharina Hellbach^3^, MD; Osman Öcal^3^, MD; Martin Loos^1^, MD; Arianeb Mehrabi^1,2^, MD

^1^Department of General, Visceral, and Transplantation Surgery, Heidelberg University Hospital, Heidelberg, 69120, Germany.

^2^Liver Cancer Center Heidelberg (LCCH), Heidelberg University Hospital, 69120 Heidelberg, Germany.

^3^Department of Diagnostic and Interventional Radiology, Heidelberg University Hospital, 69120 Heidelberg, Germany.

*These authors contributed equally to this work.

**Corresponding author information:**

Prof. Arianeb Mehrabi, MD, FICS, FACS, FEBS; Head of the Division of Liver Surgery, Department of General, Visceral and Transplantation Surgery, Heidelberg University Hospital, Im Neuenheimer Feld 420, 69120 Heidelberg, Germany.

**Email:** [arianeb.mehrabi@med.uni-heidelberg.de](mailto:arianeb.mehrabi@med.uni-heidelberg.de)

**Tel:** +4962215636223	**Fax:** +496221565781

**Supplementary Materials – Index**

**Supplementary Methods**

none

**Supplementary Results**

none

**Supplementary Appendixes**

none

**Supplementary Figures and Tables**

Figure S1. (a)

Figure S1. (b)

Figure S2. (a)

Figure S2. (b)

Figure S3. (a)

Figure S3. (b)

Figure S3. (c)

**References**

none

**Supplementary Figures and Tables

Figure S1. (a) Anastomotic leak (AL).** PTCD cholangiography image: Contrast extravasation from the bilioenteric anastomosis (arrow) confirms AL.

**(b) No AL.** PTCD cholangiography image: No contrast extravasation observed, contrast flows exclusively into the jejunal loop (arrow), effectively ruling out AL.

**Figure S2. (a) Surface leak (SL).** Postoperative CT image showing a fluid collection at the resection surface (asterisk).

*

**(b) TBL.** PTCD image demonstrating a connection between the biliary system and the fluid collection (arrow), confirming a transection surface bile leak.

**Figure S3. (a) Isolated bile duct leak (IBDL).** Postoperative CT following left Hemihepatectomy showing a large fluid collection (asterisk) adjacent to the resection area.

*

**(b) IBDL.** PTCD image demonstrating a connection (arrow) between segment V bile duct (arrowhead) and the collection, without communication to the bilioenteric anastomosis, confirming the diagnosis of an isolated bile duct leak.

**(c) IBDL.** Hepatobiliary-phase MR image (intracellular Primovist-enhanced MRI) showing contrast extravasation (arrow) into a collection, with the segment V bile duct (arrowhead) terminating in the fluid collection without any connection to the main biliary tree or the bilioenteric anastomosis.
